# An exploratory study of engagement in a technology-supported substance abuse intervention

**DOI:** 10.1186/1747-597X-5-10

**Published:** 2010-06-08

**Authors:** Nancy R VanDeMark, Nicole R Burrell, Walter F LaMendola, Catherine A Hoich, Nicole P Berg, Eugene Medina

**Affiliations:** 1 Graduate School of Social Work, University of Denver, 2148 South High Street, Denver, CO 80208, USA; 2 Colorado Social Research Associates, 3530 West Lehigh Avenue, Denver, CO 80236, USA; 3 Arapahoe House Inc., 8801 Lipan Street, Thornton, CO 80260, USA

## Abstract

**Background:**

The continuing gap between the number of people requiring treatment for substance use disorders and those receiving treatment suggests the need to develop new approaches to service delivery. Meanwhile, the use of technology to provide counseling and support in the substance abuse field is exploding. Despite the increase in the use of technology in treatment, little is known about the impact of technology-supported interventions on access to services for substance use disorders. The E-TREAT intervention brings together the evidence-based practice of Motivational Interviewing and theories of Persuasive Technology to sustain clients' motivation to change substance use behaviors, provide support for change, and facilitate continuity across treatment settings.

**Methods:**

This study used descriptive statistics, tests of statistical significance, and logistic regression to explore the characteristics and perceptions of the first 157 people who agreed to participate in E-TREAT and the predictors of their active engagement in E-TREAT services. In addition, responses to open-ended questions about the participants' experiences with the intervention were analyzed.

**Results:**

The data reveal that clients who engaged in E-TREAT were more likely than those who did not engage to be female, have children and report a positive relationship with their recovery coach, and were less likely to have completed treatment for a substance use disorder in the past. A majority of people engaging in E-TREAT reported that it was helpful to talk with others with similar problems and that the program assisted them in developing a sense of community.

**Conclusions:**

The authors conclude that technology-assisted interventions hold promise in expanding access to treatment for substance use disorders especially for women and parents. Further, the characteristics of the relationship with a coach or helper may be critical to engagement in technology-supported interventions. Additional investigation into ways technology may be useful to enhance treatment access for certain subgroups is needed.

## Background

Nationally, the demand for substance use disorder treatment in publicly funded systems far exceeds the available capacity. In 2008, over 20 million people who needed treatment for alcohol or illicit drug abuse or dependence were unable to access this needed service [1]. As a result of this gap between treatment demand and capacity, providers often establish waiting lists. Moreover, the motivation of people in need of substance use disorder treatment often fluctuates, resulting in lack of initiation of treatment and dropouts early in treatment.

Motivational Interviewing [2,3] has demonstrated effectiveness in improving engagement and retention in substance use disorder treatment by tailoring interventions to each individual client's readiness to change. Nevertheless, motivational interviewing relies on individualized, face-to-face interactions that are often neither economically nor geographically sustainable. With similar aims to Motivational Interviewing, Persuasive Technology combines intentional persuasion practices with computing technology to motivate and assist people in changing a target behavior [4], suggesting that computer applications may be useful to assist in increasing motivation to change substance use.

Over the past decade, the use of online counseling to address a variety of behavioral health problems has surged. Specifically, in the area of substance use disorder treatment, technology-assisted programs have shown potential to enhance treatment engagement and retention among pregnant cocaine abusers [5,6], disaster survivors with mental health and substance use disorders [7], and problem drinkers [8-11]. Researchers have proposed that computer- and internet-based interventions have potential to close the gap between treatment need and capacity by reaching populations that have not traditionally participated in treatment [12,13] and improving overall treatment outcomes [14]. Findings from these studies suggest that technology-assisted treatment for substance use disorders may aid populations with significant barriers in accessing treatment, such as parents, people who are employed, and older individuals, to seek help for their substance-related problems [15,16]. E-TREAT, a technology-assisted intervention for individuals seeking treatment for substance use disorders, uses interactive web-based technology to tailor responses to individual client readiness to change with the aim of improving treatment engagement and retention.

Tailored health interventions have been shown to be effective in engaging individuals and improving their healthy behavior [17]. In health interventions, tailoring has been defined as a manner of gathering and appraising information from an individual related to a behavior and creating an individualized response intended to meet that person's needs [18]. The use of technology to accomplish tailoring has grown over the past decade or more [19,20], though research on internet-based interventions is just emerging [21].

### E-counseling and support for substance abuse

Over the past decade, online counseling to address problems such as smoking, depression, and anxiety disorders has increased. In a meta-analysis comparing web-based behavioral change interventions to non-web interventions from 1996 to 2003, Wantland et al. [22] reported an elevenfold increase in MEDLINE citations for "Web based therapies." Recently, Lustria et al. [20] identified 503 "Web based" health intervention studies as potentially relevant for their meta-analysis, but of those found, only thirty "...clearly reported using computer-assisted methods for assessment and message tailoring" (p. 158). Portnoy et al. [21] included 75 randomized controlled trials from 1988 to 2007 in their meta-analysis of computer-delivered interventions for health promotion and behavioral risk reduction. Unlike Lustria's, Portnoy's study did not use internet delivery as a selection criteria for inclusion.

More recently in the area of substance use disorder treatment, there is a growing interest in the use of web-based programs to enhance treatment engagement and retention and to improve treatment outcomes [5-8, 14, 16]. In a study of pregnant cocaine abusers, those who used online services three or more times per week were found to be two to three times more likely to participate in self-care activities and treatment than clients assigned to the usual face-to-face support [6]. In a separate study, 87% of clients offered online services with therapeutic e-mail participated in the program, and use of these services was positively associated with treatment retention [5]. Moreover, effect size comparisons showed significant improvement in outcomes for those using the web-based interventions to achieve specified knowledge and behavior change. Further, in a study of e-therapy for behavioral health using brief motivational interviewing and enhancement strategies, Ruggiero et al. [7] concluded that brief interventions can be used successfully in addition to traditional care, and these interventions have the potential to increase participation among those who may not otherwise seek care. Other researchers have suggested that the use of computer tools in conjunction with treatment of substance use disorders may be a cost-effective strategy for increasing the accessibility of services, particularly with problem drinkers [8,10,23]. King and colleagues [16] found that the use of groups delivered using videoconferencing technology in conjunction with outpatient methadone treatment rivaled face-to-face groups in terms of outcomes, and all of the clients randomly assigned to videoconferencing reported a preference for continuing treatment through videoconferencing. The participants cited convenience and privacy as the driving factors in their preference for the videoconferencing condition.

In addition to improving treatment engagement and retention, there is some evidence that computer-supported interventions have the potential to improve treatment outcomes. Carroll and colleagues [14] found that the use of a computerized cognitive behavioral program in conjunction with usual outpatient treatment for substance dependence was associated with longer periods of abstinence from drug use following treatment. Thus, the emerging evidence suggests that the use of technology-supported treatment interventions presents an important opportunity to enhance access, engagement, retention, and outcomes related to substance use disorder treatment.

### Motivational Interviewing

Based in the "Transtheoretical" model known as Stages of Change [2,24], Motivational Interviewing is a client-centered counseling approach used to enhance readiness to change by reducing ambivalence. Counselors match treatment strategies with the client's degree of readiness to change. In early stages, the counselor acts as a coach to enhance motivation to change; in later stages, the counselor acts to sustain motivation and links the client with the resources and skills needed to make and sustain behavioral changes [24,25]. Motivational Interviewing is an evidence-based approach to treatment that has been demonstrated to yield positive substance use outcomes as well as to be effective in enhancing substance use disorder treatment engagement, retention, and adherence [2,3,26-30].

### Persuasive Technology

As with Motivational Interviewing, Persuasive Technology has the "'explicit purpose' to change human attitudes and behavior"[31]. Fogg [4] has developed a useful typology that describes the use of persuasion with computing technology capabilities. Fogg identifies three categories of use in his typology. The three categories are *tool*, *medium*, and *social actor*. Computing technologies are a *persuasive tool *when they make a target behavior easier to do, lead people through a process, or perform calculations or measurements that motivate people. Computing technologies are used as a *persuasive medium *when they allow people to examine cause-and-effect relationships, provide people with motivational vicarious experiences, or assist people with the rehearsal of a behavior. Computing technologies are used as a *persuasive social actor *when they reward people with positive feedback, assist people with modeling a target behavior, or provide people with social support [4].

Research indicating that technology-mediated social support groups can foster commitment is relatively recent [32] but builds on social science investigations that date back more than three decades [33]. In the 1970 s, Short et al. [33] conducted empirical investigations of their theory that communication could convey an "awareness" of the other person - a phenomenon they named *social presence *(p.76). The ensuing stream of research has variously focused on social presence as 1) being there, with others, together; 2) being involved and known; and 3) being immediately engaged in activities [34]. Later studies have found that qualities of social presence, such as the perceived "immediacy" of the group leader, were highly related to satisfaction with services, feelings of connectedness, and perceived motivation [35,36]. Social presence and immediacy are important components of engagement and adherence that need examination in persuasive technology investigations.

Projects using persuasive technology approaches have begun to demonstrate that they support evidence-based, theoretically driven development of therapeutic human service interventions [37]. Hester and Miller [8] found empirical evidence that computer-based tools addressing alcohol abuse supported increased motivation and reduced harm related to substance use. They suggested further that the key to the effectiveness of those tools might be their personalized feedback, or the degree to which the program is tailored to individuals [38]. Thus, persuasive technologies and motivational interviewing have the common aim of personalizing messages with the goal of changing behavior.

### E-TREAT Approach

Blending the principles of Motivational Interviewing and persuasive technologies, the E-TREAT intervention was designed to sustain motivation to change in individuals with substance use disorders who were waiting for treatment or in transition between treatment services. Specifically, the goals of the intervention are to provide the information, support, and encouragement needed to sustain motivation during the time that an individual is waiting to enter treatment and during transitions from one level of care to another. Although the goal of the intervention is to bolster engagement in ongoing treatment rather than replace treatment, it is expected that some individuals will choose not to enter treatment for a variety of reasons, including continued ambivalence about recovery and remission of symptoms without treatment.

Based on Alemi et al.'s [5] suggestion that clients be provided with the option of having face-to-face contact with a clinician, E-TREAT includes recovery coaches to conduct the initial face-to face assessment, assist clients in orientation to the computer resources, and orchestrate access to the variety of electronic resources available through the E-TREAT intervention. Recovery coaches, receiving supervision in Motivational Interviewing, provide personalized coaching and support, individualized feedback, and tailored motivational messages to clients using a range of technologies, including telephone, text messages, e-mail, and web-based communication. Individualized messages offer worksheets, internet resources, and suggestions about sustaining recovery efforts, such as exploring ambivalence about entering treatment, averting destructive communication patterns, and eliciting self-motivational statements.

The recovery coaches have access to a variety of tools. They assist each client in establishing a personalized home page reflecting the client's recovery-related and other interests. They initiate electronic reminders and facilitate group discussions via web and telephone conferencing. Group discussions cover a range of topics: exploring ambivalence about treatment and recovery, preparing for treatment, acquiring benefits, and developing healthy support systems. The Change Gauge, a web-based assessment tool, allows clients to assess their readiness to change their substance use behaviors using the University of Rhode Island Change Assessment [39] and provides information and suggested interventions based on their stage of readiness to change. For example, an individual in a precontemplative stage of change is provided with exercises to assist in examining the pros and cons of continued use, while an individual in a maintenance stage is provided with links to online recovery support resources. As a result, recovery coaches are able to tailor their communications to the client's readiness for change. Although clients can continue to use E-TREAT resources throughout treatment, the E-TREAT intervention is expected to assist during a period ranging from four to twelve weeks while the individual is waiting to enter a treatment program or during transitions between treatment settings. Clients are discharged from the E-TREAT intervention when they complete all treatment within the parent treatment center.

This exploratory study describes characteristics of the first 157 people seeking treatment for substance use disorders who were enrolled in the E-TREAT intervention, and examines the characteristics that predict their active engagement in the E-TREAT intervention. It further presents the participants' perceptions of the strengths and weaknesses of the intervention.

## Methods

### Intervention setting

E-TREAT is delivered through a large multimodality treatment center serving metropolitan Denver, Colorado since 1975. E-TREAT is part of a larger continuum of substance use disorder treatment services that includes social model detoxification, as well as short-term intensive residential, transitional residential, and intensive and non-intensive outpatient services. The organization receives funding from a wide variety of private and public sources, and provides detoxification and treatment services to individuals who are referred by a range of sources, including self-referrals, family members, insurance companies, probation and parole officers, child welfare agencies, hospitals, community service agencies, homeless shelters, and twelve-step programs.

Of the adults entering residential or outpatient treatment (excluding those receiving detoxification only) within the organization in 2008, 62% were male; the average age of the clients was 33 years, with a range from 18 to 78 years. Fifty-four percent of the clients reported alcohol as their primary drug, with 16% reporting crack or cocaine, 14% reporting marijuana, 12% reporting methamphetamines or amphetamines, and the remaining 4% reporting other substances as their drug of choice. Those served were predominately White, non-Hispanic (59%); 22% identified themselves as Hispanic, 12% identified themselves as Black or African American, 4% reported as other non-Hispanic, 2% reported as American Indian, and 1% identified with another race or ethnic group.

### Procedures

Clients who were determined by the agency's screening and intake department to be eligible for residential or outpatient treatment for substance use disorders within the parent organization and those transitioning from residential to outpatient treatment were screened for eligibility for E-TREAT. E-TREAT eligibility criteria were: (1) meet diagnostic criteria for substance abuse or dependence, (2) be over the age of 18, (3) be scheduled for residential or outpatient services, and (4) be willing to participate in an electronic treatment intervention designed to supplement traditional treatment.

Basic information about the E-TREAT clinical and research protocol was provided to eligible clients; if a client expressed interest, the intake department offered to transfer the call to an E-TREAT recovery coach who answered additional questions and scheduled a face-to-face meeting. If the client expressed an interest but chose not to speak to a recovery coach immediately (or a recovery coach was not available to take the call), the intake department informed the client that an E-TREAT recovery coach would contact him or her at a later time. Clients in the agency's residential programs who would soon be transitioning into outpatient programs also were recruited. The recovery coaches attended the intensive residential group meetings monthly and presented information about E-TREAT. Residential clients who expressed interest in E-TREAT and met the eligibility criteria were enrolled. Of the 882 clients who were available to be screened by intake staff or recovery coaches for E-TREAT eligibility, 157 were eligible and agreed to participate in the program and study, 78 were determined to be ineligible, 427 declined referral for eligibility screening or, once screened, declined to participate in either the program or study, and 220 could not be contacted for screening.

At the face-to-face meeting, the recovery coach reviewed the treatment consent form and the evaluator secured informed consent to participate in the evaluation of E-TREAT. If the client consented to participate in E-TREAT, the recovery coach conducted a short clinical assessment and assisted the client in accessing the E-TREAT resources by logging on to the E-TREAT website and creating a password. A research assistant collected baseline evaluation data that included demographic characteristics, alcohol and drug use information, and a motivational assessment. Follow-up interviews were conducted at three months after baseline in the project to assess changes in outcomes and perceptions of the effectiveness of the program. Western Institutional Review Board approved the procedures, and continues to oversee human research protections associated with the study.

### Measures

#### Substance use and related problems

In response to the Government Performance and Results Act (GPRA), the Center for Substance Abuse Treatment developed an instrument to collect information on substance use and related problems among grantee programs. Administration of this instrument, which is required by the funding agency [40], took place at baseline and 3 months following baseline. The domains covered in this instrument include alcohol and drug use and consequences, family and living conditions, employment and income, involvement in the criminal justice system, and physical and mental health. Although not validated itself, the GPRA measure comprises items from measures with established reliability and validity and is widely used for data collection in publicly funded substance abuse treatment settings.

#### Motivation for treatment

The Treatment Motivation Questionnaire (TMQ) [41] was administered during the baseline interview to assess participants' reasons for entering treatment. This 25-item questionnaire has been used with adults with alcohol and drug dependence and mental health problems to assess motivation to enter treatment. The measure has demonstrated test-retest reliability and internal consistency ranging from .70 to .98 [41], as well as construct validity with motivation and retention in treatment [41,42]. The measure assesses perceptions about how participants might feel when entering treatment, and perceptions about why they sought treatment, on a scale from 1 to 7 with "1 = not at all true" and "7 = very true." This questionnaire contains internal and external motivation subscales.

#### Services received by participants

Recovery coaches completed the E-TREAT Recovery Coach Daily Contact Log in order to track every contact with participants. This log was developed by the project team to track the number of minutes spent with clients through face-to-face contact, e-mail, telephone calls, text messages, instant messaging, online groups, or the bulletin board.

#### Social presence, immediacy, and satisfaction

The participant questionnaire also included Short et al.'s [33] overall social presence measure, which uses a semantic differential technique of paired items such as cold-warm, unsociable-sociable, impersonal-personal to measure the experience and perception of social presence by an individual in a particular medium. These items are each rated from 1 to 7 and then totaled to give an overall social presence score. A second set of ten items originally developed by Gunawardena and Zittle [35], and adapted by Richardson and Swan [36], was used to measure the perceived "immediacy" of E-TREAT. Immediacy is defined as a set of attitudes that participants form about service availability, accessibility, and helpfulness (e.g., "I feel comfortable using E-TREAT to communicate with others") based on the perceived behavior, in this case, of the Recovery Coach. Richardson and Swan [36] found that these perceptions were strongly associated with overall satisfaction and motivation. Three additional items originally used by Richardson and Swan [36] also were included. One measures overall service satisfaction, and the other two perceptions of the quality of the program (e.g., "The programming that took place in E-TREAT was of the highest quality"). All items are rated on a five point scale with "1 = strongly agree" and "5 = strongly disagree."

#### Internet use

An instrument assessing each participant's ability and experience with computer use was administered by the recovery coach during the initial phone call with the client.

### Analysis

Descriptive statistics were produced for all participants who agreed to participate in E-TREAT and completed a baseline interview. Using the definition of engagement established by the Washington Circle for public sector substance abuse treatment systems [43], participants were identified as either "Engagers" or those who with three or more service contacts with E-TREAT or "Non-Engagers", those who had two or fewer service contacts. Contact was defined as communication with an E-TREAT recovery coach or use of E-TREAT resources such as e-mail, instant messaging, or telephone groups. (Contacts did not include the initial face-to-face enrollment session or unreturned e-mails sent by the recovery coaches.)

One-way analysis of variance (ANOVA) and Pearson chi-square tests were conducted to compare the characteristics of these two groups. Subsequently, two sequential logistic regressions were completed, introducing the client and service variables that were significantly different between Engagers and Non-Engagers as predictor variables and engagement in the E-TREAT intervention as the dependent variable.

## Results

### Demographic characteristics of participants enrolled in E-TREAT

The 157 individuals who agreed to participate in E-TREAT during its first 10 months of operation were nearly evenly split in gender (52.2% female) and ranged in age from 18 to 65 years, with an average age of 36.6 (*SD *= 9.7) years. Reflecting the larger treatment population in the agency, the majority of individuals entering E-TREAT were White (75.8%). Additionally, 29.3% of the individuals entering E-TREAT reported their ethnicity to be Hispanic. Nearly two-thirds of the sample were unemployed (61.8%); 54.1% had been to college or technical/vocational school. Participants' average monthly income was $698 (*SD *= 14.0) ranging from no income to $13,000 per month.

Approximately two-thirds of the participants (63.7%) reported use of alcohol in the 30 days prior to the baseline interview, 24.2% reported use of cocaine, 19.7% reported use of marijuana, and 12.1% reported use of methamphetamines. Over two-thirds (68.2%) of the sample had children, and 38.2% were on probation or parole at the time they entered E-TREAT. Nearly three-quarters (70.7%) of the participants reported having internet access at the time they entered E-TREAT; 59.5% of this group reported internet access in their homes. Table 1 provides detailed demographics for the sample.

**Table 1:** Demographic characteristics of participants enrolled in E-TREAT (*N *= 157)

Variable		
**Age (*M*, *SD*)**	36.6	9.7
**Monthly income from wages (*M*, *SD*)**	698	14.0
**Gender (*n*, %)**		
Male	74	47.1
Female	82	52.2
Transgendered	1	1.6
**Ethnicity (*n*, %)**		
Hispanic	46	29.3
**Race (*n*, %)**		
White (non-Hispanic)	99	63.1
White (Hispanic)	20	12.7
Black	12	7.6
American Indian	24	15.3
Other	2	1.3
**Employed (*n*, %)**		
Employed	60	38.2
**Education (*n*, %)**		
12 years or less	72	45.9
Some college	50	31.8
Bachelors degree or higher	20	12.7
Vocational program	15	9.6
**Living (*n*, %)**		
Shelter	7	4.5
Street/Outdoors	1	1.6
Institution	3	1.9
Housed	146	93.0
**Used substance in past 30 days (*n*, %)**		
Alcohol	100	63.7
Cocaine	38	24.2
Marijuana	31	19.7
Methamphetamines	19	12.1
**Days substance use in the past 30 days (*M*, *SD*)**		
Alcohol	7.0	9.1
Cocaine	2.1	5.6
Marijuana	1.9	5.6
Methamphetamines	.85	3.0
**Children (*n*, %)**		
Yes	107	68.2
**Probation/Parole (*n*, %)**		
Yes	60	38.2
**Internet access (*n*, %)**		
Yes	111	70.7
**Of those with Internet (*n *= 111) where accessing (*n*, %)**		
Friend/Family	8	7.2
Home	66	59.5
Public access	24	21.6
Work	2	1.8
Missing	11	9.9
**Treatment Motivation (*M, SD*)**		
External	2.89	1.55
Internal	5.94	.94

### Treatment Motivation Questionnaire

There were 156 clients who completed the TMQ at baseline. At that time, the clients' mean scores were higher on the internal motivation scale (*M *= 5.94, SD = 1.55) than on the external motivation scale (*M *= 2.89, SD=.94), indicating that their reasons for entering treatment were more often internal. Table 1 includes baseline treatment motivation scores on the internal and external motivation subscales.

### Characteristics of participants who engaged in E-TREAT

Table 2 depicts the results from the ANOVA and Pearson chi-square tests comparing E-TREAT Engagers to Non-Engagers. Out of 157 clients, 39.5% (62) were Engagers, while the remaining 60.5% (95) were Non-Engagers (had two or fewer contacts). The findings demonstrate that Engagers were more often women and more likely to have children than Non-Engagers. Furthermore, E-TREAT Engagers were using marijuana more often in the prior month and less often had prior treatment for substance use disorders than Non-Engagers.

**Table 2:** Comparison of E-TREAT Engagers and Non-Engagers at baseline

Variable	Engaged *n *= 62	Not Engaged *n *= 95	Test Statistic (*df*)	*p-*value
**Age (*M*, *SD*)**	37.6 (8.3)	36.0 (10.6)		.313
**Gender (%)**			*X*^2 ^= 6.178^a ^(2,1)	.035
Male	35.5	54.7		
Female	62.9	45.3		
Transgender	1.6	0		
**Ethnicity (%)**				
Hispanic	37.1	24.2		.061
**Race (%)**				
White (non-Hispanic)	56.5	67.4		.112
White (Hispanic)	14.5	11.6		.380
Black	8.1	7.4		.551
American Indian	16.1	14.7		.491
Other	1.6	1.6		.635
**Monthly income from wages (*M, SD*)**	909.2 (1918.4)	561(906)		.128
**Used substance in past 30 days (%)**				
Alcohol	71.0	58.9		.086
Marijuana	19.4	20.0		.546
Methamphetamines	11.3	12.6		.505
Cocaine	29.0	21.1		.171
**Days substance use in the past 30 days (*M, SD*)**				
Alcohol	8.6 (9.6)	5.9 (8.6)		.069
Marijuana	3.0 (7.7)	1.2 (3.5)	*F *= 3.955^a ^(1,155)	.048
Methamphetamines	.82 (3.3)	.86 (2.8)		.934
Cocaine	2.2 (5.3)	2.0 (5.8)		.865
**Previous treatment (%)**				
SUD Treatment	33.9	52.6	*X*^2 ^= 5.330^b ^(1,1)	.016
MH Treatment	25.8	20.0		.254
**Employment (%)**				.525
Employed	38.7	37.9		
Unemployed	61.3	60.0		
**Education (%)**				.930
12 years or less	43.5	47.4		
Some college	32.3	31.6		
Bachelors degree or higher	12.9	12.6		
Vocational program	11.3	8.4		
**Living situation (%)**				.437
Shelter	1.6	6.3		
Street/Outdoors	0	1.1		
Institution	1.6	2.1		
Housed	96.8	90.5		
**Social connectedness (%)**				
Yes	91.9	93.7		.453
**Children (%)**				
Yes	80.6	60.0	*X^2^*= 7.367^b ^(1,1)	.005
**Parole/Probation (%)**				
Yes	35.5	40.0		.345
**Internet access (%)**				
Yes	75.8	67.4		.101
**Where accessing internet (%)**			.613	
Friend/Family/Work	13.0	7.4		
Home	65.2	66.7		
Public access	21.7	25.9		
**Entered Treatment (%)**				
Yes	53.2	42.1		.115
**Treatment Motivation (*M, SD*)**				
External	2.9 (1.5)	2.9 (1.6)		.825
Internal	5.9 (1.1)	6.0 (.8)		.741

^a ^one way analysis of variance

^b ^Pearson chi square

### E-TREAT services received

Table 3 describes the various types of services used by those who engaged in E-TREAT and those who did not engage in E-TREAT. On average, individuals engaged in E-TREAT had 7.6 (*SD *= 5.3) contacts with their recovery coach, totaling an average of 213.3 (*SD *= 117.8) minutes. The majority of participants engaged in, on average, 3.8 (*SD *= 4.7) types of services combining any of the following interventions: phone, e-mail, bulletin board, text messaging, instant messaging, or phone groups. The combination of activities does not include use of the website or the initial face-to-face visits.

**Table 3:** Services received by participants in E-TREAT

Variable	Engaged (n = 62)				Not Engaged (n = 95)				Test Statistic	*p-*value
	*M*	*SD*	Min	Max	*M*	*SD*	Min	Max	(*df*)	
Total contacts:	7.6	5.3	3	23	1.1	.78	1	2	*F *= 74.770^a^(1,110)	.000
Total contact minutes:	213.3	117.8	55	555	101.1	34.1	10	197	*F *= 75.890^a ^(1,154)	.000
Face-to-face minutes:	111.2	61.92	30	320	88.6	25.9	30	180	*F *= 9.628^a ^(1,149)	.002
Phone contacts:	3.7	3.6	0	18	.8	.6	0	2	*F *= 82.784^a^(1,155)	.000
Phone minutes:	35.3	35.7	0	159	4.6	8.3	0	45	*F *= 64.782^a^(1,155)	.000
Electronic contacts:	3.8	4.7	0	22	.2	.5	0	2	*F *= 54.642^a^(1,155)	.001
Electronic minutes:	105.6	94.0	3	465	15.1	17.6	0	72	*F *= 83.719^a^(1,155)	.000

^a ^one way analysis of variance

### Ratings of service immediacy

Preliminary data on perceptions of the immediacy of the service, based on the ten-item Richardson & Swan scale [36], were collected from a subset of participants who were located three months following baseline, and are found in Table 4. Two-thirds (65.9%) of participants who reported that they had participated in E-TREAT found it to be effective in helping them to talk with others who had similar problems, 53.7% found that the recovery coaches created a feeling of community, and 52.4% reported feeling comfortable using the technology to communicate with others.

**Table 4:** Service immediacy ratings at follow-up (*N *= 82)

Statement	Participants who agreed or strongly agreed		Participants who reported that they did not use the service	
	*n*	%	*n*	%
E-TREAT is an excellent way to talk with others who have the same problems I do	54	65.9	16	19.5
I felt comfortable using E-TREAT to communicate with others	43	52.4	25	30.5
I felt comfortable introducing myself in a group	33	39.0	31	37.8
Introductions to other participants helped me form a sense of community	29	35.4	34	41.5
The recovery coach created a feeling of community	44	53.7	22	26.8
I felt comfortable participating in group discussions	23	28.0	40	48.8
The recovery coach facilitated discussion in the group	24	29.3	42	51.2
I felt comfortable interacting with other group participants	30	36.6	36	43.9

### Overall social presence, overall satisfaction, and recovery coach satisfaction

Several other types of preliminary data were gathered from the participant questionnaire. These data, summarized in Table 5, include mean comparisons of Engagers and Non-Engagers on the Short et al. social presence scale [33], and mean scores on the participant overall service and recovery coach satisfaction items. These data indicate that overall satisfaction with recovery coaches was significantly higher among individuals who engaged in E-TREAT as compared with those who did not engage.

**Table 5:** Comparison of engaged and not engaged on social presence, overall satisfaction, and recovery coach satisfaction at follow-up

Variable	Engaged				Not Engaged				Test	*p-*value
	*M*	*SD*	*Min*	Max	*M*	*SD*	Min	Max	Statistic (*df*)	
Overall social presence (*n *= 27, 34)	4.3	.86	2	5	4.2	.99	1	5		.643
Overall E-TREAT (*n *= 29, 30)	3.9	1.0	1	5	4.0	1	1	5		.895
Overall recovery coach (*n *= 30, 30)	4.4	.56	3	5	3.8	.94	2	5	*F *= 9.122^a ^(1,58)	**.004**

^a ^one-way analysis of variance

### Responses to open-ended questions

The 82 E-TREAT participants completing a three-month follow-up interview were asked open-ended questions regarding their experiences with the intervention. Participants were first asked if they were happy with the E-TREAT program overall. More than half responded that they were; the main theme that emerged was the support that the participants received from the program. One participant commented that it was "good to have different networks of support." Other responses to this question indicated that participants found E-TREAT helpful, informative, accessible, and convenient. Participants also were asked which part of E-TREAT was the most helpful. Many mentioned the interactions with the recovery coaches and other online users as being particularly helpful. One participant responded that she was able to "talk to different people going through the same thing and they would understand."

Conversely, participants were asked which part of E-TREAT was the least helpful to them. Computer accessibility and ease of use emerged as the primary and secondary themes for this question. Some participants had computer access initially when they enrolled in E-TREAT services and then lost it. Other participants experienced log-on problems that they were never able to resolve. Finally, respondents that reported using the E-TREAT website "a few times per month or never" were asked to explain why they chose not to use E-TREAT services. The primary theme that emerged for this question was accessibility, followed by being too busy and/or not having enough time. Some respondents entered a residential treatment program where they did not have computer access. Others stated that they were just too busy with work or spending time with family.

### Predictors of Engagement

Two sequential logistic regressions were conducted to further examine the predictors of engagement in E-TREAT. The variables listed in Table 2 that were found to be significantly different in the ANOVA and Pearson Chi Square tests comparing Engagers and Non-Engagers were grouped together as client variables and examined as predictors of engagement. The two service variables that were significantly different between Engagers and Non-Engagers at the three-month follow-up point and not included in the definition of engagement- overall recovery coach rating (*F *= 9.12, *df *= 1,58, *p *= .004) and number of minutes spent at the initial face-to-face meeting (*F *= 9.628, *df *= 1,149, *p *= .002) - were grouped together and examined as service-level predictors of engagement in E-TREAT.

The first logistic regression that included the four client-level variables demonstrated that, when considered together, the client-level variables significantly predicted engagement in E-TREAT (*X*^2 ^= 18.38, *df *= 4, *N *= 156, *p *= .001). The odds ratios shown in Table 6 demonstrate that people with children were more than twice as likely to engage in E-TREAT as clients who did not have children, and that women were nearly two times as likely as men to engage.

**Table 6:** Logistic regression examining client variables as predictors of engagement *N *= 156

Variable	Wald (*df*)	Odds ratio	95% Confidence Interval	
Gender	3.340 (1)^a^	1.913	.954	3.837
Children	4.403 (1)^b^	2.310	1.057	5.049
Marijuana use	3.391 (1)^c^	1.067	.996	1.142
Prior treatment	3.451 (1)^d^	.517	.258	1.037

^a ^*p *= .068; ^b ^*p *= .036; ^c ^*p *= .066; ^d ^*p *= .063

The second logistic regression that included the two service variables collected at the three-month follow-up interview also was significant, demonstrating that, when considered together, overall recovery coach rating and minutes of face-to-face contact with the recovery coach in the initial session were found to predict engagement in E-TREAT (*X*^2 ^= 11.07, *df *= 2, *N *= 60, p = .004). As shown in Table 7, clients who rated their recovery coach more highly were three times more likely to engage in E-TREAT than those who gave their recovery coach low ratings.

**Table 7:** Logistic regression examining service variables as predictors of engagement *N *= 60

Variable	Wald (*df*)	Odds ratio	95% Confidence Interval	
Recovery coach	7.117 (1)^a^	3.141	1.355	7.282
Face to face	1.868 (1)^b^	1.009	.996	1.023

^a ^*p *= .008; ^b ^*p *= .172

## Discussion

Although the proportion of clients enrolled in E-TREAT who actively engaged in the intervention is disappointing, the findings presented here are from the initial set of clients receiving an intervention that was quite innovative and in the early stages of refinement. It is possible that poor technology access, including earlier-than-anticipated entry into treatment where access to technology was limited, created a barrier to active engagement that could be mediated in future projects. Nevertheless, the findings from this exploratory study of engagement in a technology-supported intervention suggest that a range of clients entering and leaving treatment are willing to participate in treatment and support activities using technology.

Consistent with studies of computer use [44] and engagement in e-therapy for alcohol problems [15], women were more likely than men to engage in E-TREAT services. Theories about the importance of relationships in women's recovery from substance use disorders [45], coupled with participants' responses that building new networks of support contributed to satisfaction with E-TREAT, may suggest that women found the social aspects of the technology-supported intervention particularly compelling. Other explanations such as avoiding the gender-specific stigma related to substance use disorders among women may also account for the gender differences detected. These findings suggest that women may find particular benefit in technology-mediated interventions for substance use disorders that should be considered in the design of these interventions.

The present study also points toward the potential of technology to address critical barriers in treatment access. In this study, parents were more likely than non-parents to engage in E-TREAT services. As discussed by King et al. [16], participants with children may have been more inclined to use E-TREAT resources than those who did not have children because doing so allowed them to participate in services without the child-care barriers inherent in traditional treatment settings. Nonetheless, one of the reasons given for nonparticipation in E-TREAT was family demands, which raises questions about the degree to which treatment motivation and access barriers are intertwined.

The relationship between not having had substance abuse treatment prior to the baseline interview and engagement in E-TREAT is interesting. Although this finding does not appear to be related to severity of substance use disorder or source of study recruitment, it may reflect a skepticism that this type of intervention will be helpful among those who have failed to remain in recovery following prior treatment. As research in the use of technology to engage and treat people with substance use disorders proceeds, it will be important to explore differences in treatment history as they relate to the use of technology to improve entry into treatment.

The findings related to recovery coach contributions to perceptions of service availability, helpfulness, and accessibility/immediacy parallel earlier findings [36], where such perceptions were key to participant service use and satisfaction. The overall influence of the recovery coach in fostering engagement is significant when compared to social presence and satisfaction with services, and also is consistent with prior findings. However, the lack of difference in measurable social presence between Engagers and Non-Engagers differs from most prior literature and cannot be explained here. It is possible that the initial attention of the recovery coach and any immediate service access that followed provided more benefit than participants had hoped to have in the period of time reported here, thus leading them to favorable judgments about E-TREAT even at very low levels of contact (2 or fewer contacts). However, the divergence of the findings from prior studies suggests the need for further investigation.

The findings from the open-ended questions reveal, as King et al. [16] found, that challenges with the technological aspects of the intervention were significant. The findings suggest that, to be effective, technology-based interventions must address ease of use and provide ample technological support to users. Alemi et al. [5] and King et al. [16] have both identified barriers to internet access as significant impediments to clients' ability to participate in technology-supported treatment. King et al. [16] found that only 20% of the substance-abuse clients approached to participate in a web-based adjunctive treatment intervention had access to the internet, and that many of those who had internet access had limited knowledge of technology. In King et al.'s study, although some participants brought their computers to a clinic for assistance, no field support was offered. The present study finds that internet access at the time of program entry itself was not associated with engagement; however, the responses to the open-ended questions suggest that access to technology changes during the course of treatment and that access is an important factor in successful implementation of technology-supported interventions. Thus, in order to maximize effectiveness of such interventions, treatment providers should plan to provide ongoing technology assistance to clients. This includes access to technology during residential treatment stays.

Because some participants in the present sample entered E-TREAT prior to a treatment experience, while others entered during a treatment episode, it is not possible to discern the impact of the E-TREAT intervention on subsequent entry into treatment for substance use disorders or to distinguish between the benefits for clients at different stages of treatment. In addition, the preliminary nature of the study precluded both examination of the subtypes of electronic services that may be useful to clients with differing characteristics and examination of service outcomes. Nonetheless, these are important issues to be explored in the future.

Although the findings from the present study suggest potential for the use of technology to enhance substance use disorder treatment resources, it had many limitations. It was designed as an exploratory study and intended to capture experiences with the implementation of a pilot intervention that was in continual refinement throughout the data collection period. Although the project was embedded in a multimodality treatment agency with a large pool of potentially eligible participants, the sample was not systematically recruited from all treatment inquiries. In circumstances where monthly recruitment goals had been attained, follow-up on eligibility screening was not consistently conducted. Further, the sample included both individuals waiting for treatment as well as those transitioning out of residential programs. For these reasons, caution must be used in generalizing the findings to all individuals entering treatment. Finally, due to the number of hypothesis tests conducted, the risk for a Type I error is inflated; therefore, the significant test results reported should be interpreted with caution.

Due to the early development of the intervention, the limitations in sampling and the exploratory nature of the project as a whole, this study is intended to shape future intervention and research efforts in an area of service development that is in its infancy, rather than to contribute definitive findings. Despite the exploratory nature of this study, it provides support for the rapidly growing body of literature suggesting that technology-assisted interventions have the potential to enhance the effectiveness of substance use disorder treatment by providing an alternative means of presenting information, support, and encouragement to treatment-seeking individuals.

## Conclusions

Although the overall engagement rate in E-TREAT of 40% is disappointing for an intervention designed to increase service accessibility, there are a number of explanations that give reason for optimism about the potential of this type of intervention. First, these data describe the first participants in a newly developed pilot intervention. The intervention was being developed and refined as the first clients were enrolled, yet nearly two-thirds of those who engaged in the intervention found it an "excellent" way to seek support from others in similar circumstances. Coupled with the responses to the open-ended questions suggesting the potential of the intervention to reduce some of the access barriers inherent in traditional treatment, it appears that technology-supported treatment has the potential to play an important role in enhancing and expanding the availability of substance use disorder treatment particularly with populations such as women and parents. Further the importance of a helpful and immediate relationship with a coach or other helper should not be overlooked in the design of technology-assisted interventions. Given the sizeable gap between the number of individuals seeking treatment for substance use disorders and treatment capacity [1], technology-facilitated interventions appear to offer important opportunities to for policy makers and treatment program administrators to expand service availability and reduce barriers to access of critical health services.

## Competing interests

The authors declare that they have no competing interests. The E-TREAT intervention was developed with public support and is available for use by other behavioral health providers.

## Authors' contributions

NRV conceptualized study design, drafted the manuscript, and assisted in data analysis. NRB completed the quantitative data analysis, drafted portions of the manuscript, and reviewed drafts of the manuscript. WFL assisted in the conceptualization of the study and drafted portions of the manuscript. CAH assisted in the conceptualization and drafting of the manuscript. NPB completed the analysis of the open-ended questions, drafted portions of the manuscript, and reviewed drafts. EM drafted intervention portions of the manuscript and reviewed drafts. All authors read and approved the final manuscript.
